# Global drivers of variation in cup nest size in passerine birds

**DOI:** 10.1111/1365-2656.13815

**Published:** 2022-10-02

**Authors:** Karina Vanadzina, Sally E. Street, Susan D. Healy, Kevin N. Laland, Catherine Sheard

**Affiliations:** ^1^ School of Biology University of St Andrews St Andrews UK; ^2^ Department of Anthropology Durham University Durham UK; ^3^ School of Earth Sciences University of Bristol Bristol UK

**Keywords:** differential allocation hypothesis, museum collections, nest size, parental investment, passerine nests, predation threat

## Abstract

1. The size of a bird's nest can play a key role in ensuring reproductive success and is determined by a variety of factors. The primary function of the nest is to protect offspring from the environment and predators. Field studies in a number of passerine species have indicated that higher‐latitude populations in colder habitats build larger nests with thicker walls compared to lower‐latitude populations, but that these larger nests are more vulnerable to predation. Increases in nest size can also be driven by sexual selection, as nest size can act as a signal of parental quality and prompt differential investment in other aspects of care. It is unknown, however, how these microevolutionary patterns translate to a macroevolutionary scale.
2. Here, we investigate potential drivers of variation in the outer and inner volume of open cup nests using a large dataset of nest measurements from 1117 species of passerines breeding in a diverse range of environments. Our dataset is sourced primarily from the nest specimens at the Natural History Museum (UK), complemented with information from ornithological handbooks and online databases.
3. We use phylogenetic comparative methods to test long‐standing hypotheses about potential macroevolutionary correlates of nest size, namely nest location, clutch size and variables relating to parental care, together with environmental and geographical factors such as temperature, rainfall, latitude and insularity.
4. After controlling for phylogeny and parental body size, we demonstrate that the outer volume of the nest is greater in colder climates, in island‐dwelling species and in species that nest on cliffs or rocks. By contrast, the inner cup volume is associated solely with average clutch size, increasing with the number of chicks raised in the nest. We do not find evidence that nest size is related to the length of parental care for nestlings.
5. Our study reveals that the average temperature in the breeding range, along with several key life‐history traits and proxies of predation threat, shapes the global interspecific variation in passerine cup nest size. We also showcase the utility of museum nest collections—a historically underused resource—for large‐scale studies of trait evolution.

## Graphical abstract

This study investigates global correlates of nest size in 1117 cup‐nesting passerine species. Using phylogenetic comparative methods, it demonstrates that, when controlled for body size and phylogeny, the outer volume of nests is linked to both temperature and predation threat while the inner volume is correlated solely to clutch size.

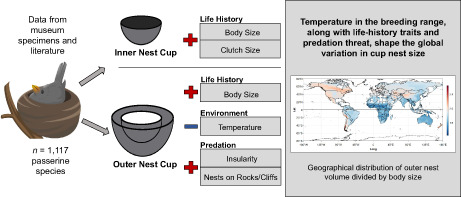

## 1 INTRODUCTION

Nests are built by a variety of organisms, including insects, fish, reptiles, birds and mammals. They encompass a wide array of shapes and sizes, and represent an important aspect of parental care (Barber, 2013; Hansell, 2000, 2005). In particular, many avian species build a nest to ensure a safe and stable microclimate for the development of their young (Hansell, 2000). A well‐constructed nest that protects eggs from extrinsic factors such as predation or cold, heat and humidity can conserve energy for later stages of parental care and therefore result in healthier offspring and higher reproductive output (Mainwaring & Hartley, 2013; Reid et al., 2000). Intraspecific variation in the size of a nest is thought to map closely to climatic conditions in the breeding range and, though often ephemeral, nest structure can be a key component in ensuring survival (Mainwaring et al., 2017). Field studies have shown that individuals of the same species build larger and more heavily insulated nests in colder environments associated with higher latitudes and altitudes (Crossman et al., 2011; Kern & Van Riper, 1984; Mainwaring et al., 2014; Rohwer & Law, 2010). Furthermore, in areas with high precipitation, nests are more porous and contain fewer absorptive materials such as fur and feathers, which can translate into an overall reduction in nest size (Heenan et al., 2015; Rohwer & Law, 2010). This link between climate and nest dimensions has been investigated primarily at an intraspecific level and within local contexts; however, it is not known whether these effects persist on a global scale (Perez et al., 2020).

The effect of climate on nest size is modulated by a range of other intrinsic and extrinsic factors, including species' life histories and the risk of predation and/or brood parasitism. Most notably, heavier species tend to build larger nests (Deeming, 2013; Heenan & Seymour, 2011; Slagsvold, 1989). Small nests physically constrain the total egg volume; by building a larger nest, species can sustain larger clutches and prevent overcrowding of chicks (Møller et al., 2014; Slagsvold, 1982, 1989). Increases in nest size, however, might be limited by the level of predation risk in the breeding environment, especially from diurnal predators that rely on visual cues. While some studies have shown that larger, more conspicuous nests are associated with higher rates of predation and brood parasitism (e.g. Antonov, 2004; Biancucci & Martin, 2010; Møller, 1990; Soler et al., 1999), others fail to find such relationship (e.g. Jelínek et al., 2015; Weidinger, 2004). On a global scale, predation threat is thought to vary across a latitudinal gradient, with the highest mortality rates found in the tropics (Ricklefs, 1969; Snow, 1978)—a trend supported by a recent meta‐analysis showing an increase in the proportion of nests lost to predators towards the equator (Matysioková & Remeš, 2022). All other things being equal, one would thus expect to find smaller nests at lower latitudes. However, this effect would, in practice, be confounded with any correlation between temperature and nest size. Furthermore, island‐dwelling populations have lower levels of predation and are associated with repeated losses of anti‐predator behaviours in a variety of animals (Beauchamp, 2004; Blumstein & Daniel, 2005; Cooper et al., 2014); nests might thus be predicted to be larger on islands compared to those on the mainland. Finally, the placement of the nest has also been shown to relate to predation—as evidenced for example by observations that species nesting above ground or in cavities are exposed to lower predation rates compared to species nesting on the ground or in the open (Martin, 1995; Matysioková & Remeš, 2022; Ricklefs, 1969)—and thus nest size would be expected to increase in hard‐to‐reach locations where detection by predators is less likely.

In addition to these environmental and life‐history drivers, nest size may also be subject to sexual selection, as individuals in better condition can construct larger nests compared to poor quality mates (Broggi & Senar, 2009; Mainwaring & Hartley, 2009; Soler et al., 1995, 2007; Tomás et al., 2013). While the use of nest size in mate choice has been documented in some bird species (e.g. Hoi et al., 1994 showed that females preferred males with larger nests in penduline tits *Remiz pendulinus*), nest size can also act as an honest sexual signal over the duration of parental care. The differential allocation hypothesis posits that parents invest more in their offspring when paired with high‐quality partners (Sheldon, 2000), but whether this phenomenon accounts for some interspecific variation in nest size, and how it might interact with other potential macroevolutionary drivers (such as climate or predation), has rarely been examined. In one phylogenetic comparative study of nest size as a post‐mating sexual signal, on 76 species of Palearctic passerines, Soler et al. (1998) found that nests were nearly twice as large and were associated with longer nestling periods in species with biparental building compared to species in which only the female builds, even though there was no associated difference in clutch size. In their interpretation, biparental builders are able to assess each other during the nest‐building stage which, on an evolutionary time‐scale, leads to bigger nests in those species because both partners signal their quality through an increased contribution to the nest. This study, however, controlled for latitude but no other environmental variables that could potentially account for the observed pattern. It also does not rule out the explanation that higher investment in nests by biparental builders simply reflects the additional energetic contribution made by the male. Given this study's limited geographical scope and comparatively small sample size, the interplay between climate, predation and the differential allocation hypothesis in driving variation in passerine nest size on global scale remains underexplored.

The order Passeriformes comprises more than 6000 species distributed across all geographical realms except for the Antarctic. The availability of life‐history information (Cornell Laboratory of Ornithology, 2020), phylogenetic data (Jetz et al., 2012) and climate conditions in breeding ranges (BirdLife International, 2019; Fick & Hijmans, 2017) makes passerines well‐suited for investigating which, if any, of these potential drivers of variation in nest size scale globally. Furthermore, while bird nests can exhibit a range of different shapes (Hansell, 2000), the majority of extant passerines construct open cup nests (Collias, 1997), meaning that their nest shape is comparable across species breeding in a variety of environmental conditions. Many of these nests have been preserved in museum collections, including in the Natural History Museum at Tring (UK), which hosts one of the largest ornithological collections in the world with more than 3300 passerine nests (Natural History Museum, 2019). Here we investigate the relative effects of climate, life‐history traits, predation and sexual selection in influencing the cross‐species variation in nest size by assembling data from 1117 species of passerines that build open cup nests across a broad geographical range. To achieve a large and ecologically diverse sample, we combine species‐level measurements from handbooks and museum specimens. Using phylogenetic comparative methods, we first focus on the outer volume of the nest (with inner volume subtracted) to reflect the total amount of nest material used, as a representation of total parental investment in the nest. If global, macroevolutionary processes were to match evidence from local, field‐based studies, we would expect increased nest size to correlate with larger parental body mass and clutch size, low temperatures, low precipitation and reduced predation threat (e.g. on islands, in inaccessible locations or in high latitudes). We then conduct separate analyses of inner cup volume, an aspect of nest ‘size’ shown to be less affected by temperature and precipitation (Mainwaring et al., 2014; Rohwer & Law, 2010). Finally, to further investigate the relationship between nest size investment and parental care evolution, we evaluate the difference in the outer nest volume between species where nests are constructed solely by a female parent or biparentally, with female‐built nests expected to be smaller. We also assess whether larger nests correlate with an increase in nestling period duration (while taking into account differences in climatic conditions within species' breeding range, nest location and body size), as a test of the differential allocation hypothesis. Taken together, these analyses evaluate the extent to which nest size, a key aspect of avian parental investment, is shaped by environmental factors, predation threat and life‐history traits at the macroevolutionary scale.

## 2 MATERIALS AND METHODS

### 2.1 Nest measurements

**FIGURE 1:**
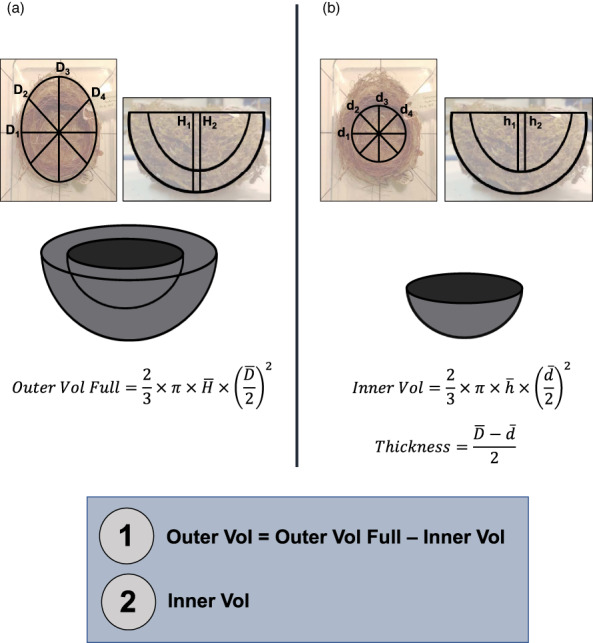
Schematic drawing of (a) outer and (b) inner nest size measurements of museum specimens. D/d, diameter; H/h, height; Vol, volume; the bar above a letter denotes the mean value of four measurements of nest diameter (D_1–4_, d_1–4_) or two measurements of nest height (H_1–2_, h_1–2_). Outer and inner nest volumes, highlighted in the blue box, are the main focus of the study. Background images of nests in courtesy of the Trustees of the Natural History Museum, London.

We obtained measurements of inner and outer nest dimensions for 1401 cup nests from 435 *Passeriformes* species from the Natural History Museum at Tring, UK, with an average of 3.16 nests/species (range 1–14). As illustrated in Figure 1, the inner and outer nest volumes were calculated using the formula for half a spheroid, as it best approximates the shape of an open cup nest (Møller, 1990). We used the average values of four measurements of nest diameter distributed equally along the circumference of the nest and two measurements of height for both the whole nest and the inner cup; the outer nest volume was obtained by subtracting the inner cup volume from the full nest volume. The nest type was identified in advance of the visit using information from the Handbook of the Birds of the World (del Hoyo et al., 2019) to exclude species that construct both domed and cup‐shaped nests in case the associated specimens were incomplete domes. The nests were measured by a single observer (KV) using a digital steel calliper with 0.02 mm accuracy, 20 cm in length (eSYNic, Ltd), in July and August 2019. Only internal dimensions were measured for specimens (*n* = 19) that appeared to be incomplete, were misshapen by the container used for museum storage or were clearly labelled as found in an enclosed habitat (e.g. a tree hole or a nest box) that might constrain the outer dimensions of the nest. No live animals were involved in this study, precluding the need for ethical approval; to better protect precious collection material, however, we took care to exclude specimens from critically endangered or extinct species.

These measurements were then supplemented with data from the Handbook of the Birds of the World (del Hoyo et al., 2019), the Birds of North America (Cornell Lab of Ornithology, 2019a), Neotropical Birds Online (Cornell Lab of Ornithology, 2019b) and the Birds of the Western Palearctic (Cramp et al., 2008). We included multiple records per species if studies could be clearly distinguished as referring to different subspecies or field sites, and averaged each nest size measurement (i.e. internal and external nest diameter and height) for each record. Nest measurements without ‘internal’ or ‘external’ qualifiers were assumed to refer to the external portion of the nest. Descriptions with values separated with ‘by’ or ‘×’ were interpreted as nest length and width (i.e. D_1_ and D_3_ in Figure 1); if three values were distinguished, the third value was assumed to be nest height. Nest ‘depth’ with no qualifiers was interpreted as external nest height, unless directly preceded by a description of inner cup diameter. As the source of nest measurements could introduce bias into our dataset, given the potential for museum nest specimens to shrink or due to differences in species composition, we included the origin of the record (literature or a museum) as a predictor of nest size in further analyses.

### 2.2 Life‐history variables and nest location

We included parental body size and clutch size as the main life‐history predictors of nest volume in phylogenetic comparative analyses. The adult body size variable represented the geometric mean of average mass for males and females, except for cases where the mass of only one sex was known. The data were sourced mainly from Dunning (2007) with additions from primary and secondary literature (comprehensive dataset for almost all bird species available from Tobias et al., 2022). Information on clutch sizes was obtained primarily from the Handbook of the Birds of the World (del Hoyo et al., 2019), complemented by the Birds of North America (Cornell Lab of Ornithology, 2019a) and the Neotropical Birds Online (Cornell Lab of Ornithology, 2019b). Using the same sources, we also collected information on the identity of the nest‐building parent as ‘female’, ‘mainly female’ or ‘both’ where available, as well as the duration of the nestling period, which was defined as the time in days from hatching until leaving the nest. We focused on the parental contribution to breeding nests and excluded constructions that form part of the courtship ritual (e.g. ‘cock’ nests built by males in some species of Sylviidae warblers, Cornell Laboratory of Ornithology, 2020). Though we identified a few species where the breeding nest was built mainly or exclusively by the male (*n* = 8), the sample was too small to incorporate this category in our analyses. We also excluded species where all care was provided by the female parent (*n* = 24) or involved assistance from juvenile or adult helpers (*n* = 62) to provide a direct comparison to the analysis by Soler et al. (1998) who included species where two parents cared for young after the nest was constructed either by both or by female alone. For both clutch size and nestling period, we used values that were highlighted as ‘mostly’, ‘usually’ or ‘typically’ characteristic of a species instead of average values, if specified. We additionally accounted for migratory behaviour due to its potential effect on reproductive traits, for example, migratory birds exhibit larger clutch sizes and shorter developmental periods compared to residential species (Jetz et al., 2008; Minias & Włodarczyk, 2020). Data on migratory behaviour were sourced from BirdLife International (2019), with ‘full migrants’ and ‘altitudinal migrants’ scored as migratory, and ‘non‐migrants’ and ‘nomads’ scored as non‐migratory.

Information on nest location was collected to provide a proxy for predation risk in the immediate vicinity of the nest. We distinguished three, non‐exclusive (i.e. treated as separate binary variables) categories of nest location based on descriptions and photos/videos primarily from the Handbook of the Birds of the World (del Hoyo et al., 2019): (1) on ground, (2) in vegetation such as in trees and in bushes and (3) on cliffs or rocks. As part of the analysis of the inner nest dimensions, we also distinguished an additional category of obligate cavity nesters, defined as always nesting in a natural or artificial cavity (*n* = 52 species). These species were excluded from analyses of the outer measurements of the nest (i.e. external diameter and height, rim thickness and outer volume) because in these cases nest dimensions might vary depending on the size of the cavity, and nest shape can diverge substantially from that of a half a spheroid. In addition to a categorical assessment of nest location, we obtained the mean height of species' nest site from the ground (m) from the minimum and maximum values provided in the Handbook of the Birds of the World (del Hoyo et al., 2019).

### 2.3 Environmental and biogeographical variables

The species range polygons were sourced from BirdLife International (2019). We included records from species' breeding or residential range (seasonal distribution codes 1 = ‘resident’ and 2 = ‘breeding season’). Only the native ranges of extant species were included in the analysis (origin distribution code 1 = ‘native’ and 2 = ‘re‐introduced’; presence distribution code 1 = ‘extant’). We obtained the coordinates of species range midpoint and determined whether this midpoint is located in the southern or northern hemisphere. This latter variable was included because species breeding in the northern hemisphere exhibit consistent differences in a range of life‐history traits, for example, shorter life spans, compared to the southern species due to the disparity in the land mass area (Scholer et al., 2020). To capture environmental variation affecting the breeding range of each species, we obtained mean temperature and precipitation from the WorldClim v.2.1 database at 10‐min resolution for 1970–2000 (Fick & Hijmans, 2017). Following Matysioková and Remeš (2022), we selected WorldClim layers that best approximated the conditions during the breeding season. For northern temperate species (range midpoint above 23.5°N, *n* = 449), we used the mean values from March to June (inclusive); for southern temperate species (range midpoint above 23.5°S, *n* = 159), we used the mean values from September to December (inclusive). For tropical species (*n* = 509), we used the mean annual values. The species range polygons were intersected with a 0.5° × 0.5° grid in ‘letsR’ r package (Vilela & Villalobos, 2015). We merged the resulting presence–absence matrix with the WorldClim layers and calculated the mean value of each environmental variable per grid cell; these values were then averaged across all cells within a species' range to obtain a mean value per species. For visualisation purposes only, we additionally generated a presence–absence matrix using a 0.25° × 0.25° grid of higher resolution.

The insularity variable was obtained by intersecting species range maps with a database of global geography (GSHHG v2.3.7, Wessel & Smith, 2017). Following Weigelt et al. (2013) and Cooney et al. (2020), we selected marine islands that exceeded 1 km^2^ but were smaller than Greenland at 2,000,000 km^2^ from full‐resolution landmass shapefiles. To test whether the reduction in predation pressure associated with island‐dwelling could differ depending on the size of the island, we additionally selected a lower, 2000 km^2^ threshold of insularity. The island layer was rasterised using a 0.5° × 0.5° grid in r package ‘raster’ (Hijmans, 2021). All cells that were covered by the island layer by a fraction >0 were counted as insular using the *getCover* argument within the *rasterize* function. The presence–absence matrix generated in the previous step was intersected with the island raster to obtain an estimate of insularity per species. Following the approach used in Cooney et al. (2020), the species was then categorised as insular if the proportion of species' range that occurred on islands exceeded 90%.

### 2.4 Phylogenetic comparative methods

We first reconstructed the ancestral state of the mean outer nest volume using *fastAnc* function in r package ‘phytools’ (Revell, 2012). The estimate represented the average value across a distribution of 1000 hypothesised topologies, drawn from the Hackett backbone of the Jetz et al. (2012) bird tree. For illustrative purposes, the evolutionary change was visualised on a single tree extracted from the full distribution in r package ‘ggtree’ (Yu et al., 2017). As we would expect the outer nest volume to be highly correlated with body size, we obtained the average Pearson correlation coefficient (*r*) between these two variables while accounting for the phylogenetic structure across the tree distribution using the *phyl.vcv* function in ‘phytools’. Finally, to validate our overall phylogenetic approach, we estimated the strength of phylogenetic signal in both traits by fitting Pagel's *λ* model (Pagel, 1999) to log‐transformed outer nest volume and body size across all trees using the *phylosig* function in ‘phytools’.

To quantify the relative importance of each predictor in determining nest dimensions, we ran Bayesian phylogenetic mixed models (BPMMs) in the r package ‘MCMCglmm’ (Hadfield, 2010); see Table S1 in Appendix S1 for the full list of models. While variation in the outer and inner nest volume remained the main focus of our analysis, we ran additional models with external and internal diameter, external and internal height, rim thickness and full nest volume as response variables. Using a smaller sample of species with available information and in which both parents care for young (following Soler et al., 1998), we assessed whether the outer nest volume correlates with the identity of nest builders (i.e. only female, mainly female or both parents). We also investigated the relationship between nest size and investment in parental care by running a model with nestling period as the response variable and the outer nest volume as one of the predictors in the reduced dataset for which the nest‐builder identity was known. Nest dimensions, clutch size, body size, height of nest site and nestling period were log‐transformed while latitude, mean annual precipitation and mean annual temperature were square‐root transformed before the analysis due to the presence of strong to moderate right skewness in the untransformed data, with all continuous variables mean‐centred and expressed in units of standard deviation. The variance inflation factor of all non‐interaction variables in all models was less than six, demonstrating that multicollinearity was not a concern in our analyses (Dormann et al., 2013). We included phylogenetic relatedness as a random effect to control for the non‐independence of traits in species that share common ancestry; to help account for phylogenetic uncertainty, we used the distribution of 1000 trees (Jetz et al., 2012) that we previously employed for the ancestral state reconstruction. As 253 species had multiple records from different populations or study sites, we included measurement record as the smallest unit of variation in our analyses. We assessed the extent of this within‐species variability by including species name as another random effect in our models following de Villemereuil and Nakagawa (2014), with the exception of the nestling period model, where the average value per species was used. This term accounted for any residual ‘between‐species’ effects that were independent of phylogeny, meaning that ‘residual variance’ corresponded to within‐species variability in our model outputs. We additionally calculated repeatability coefficients, that is, the proportion of the total phenotypic variance attributable to between‐species differences (Stoffel et al., 2017), for all types of nest dimensions in the subset of 1401 museum specimens from 435 species measured by a single observer (KV).

Following the recommendations in Hadfield (2010), we used inverse‐Wishart priors for the phylogenetic, between‐species and residual variance (*V* = 1, *ν* = 0.02) and diffuse normal priors for fixed effects (mean 0, *V* = 10^10^). We first conducted a dummy run of 1.2 × 10^5^ iterations on a single tree with a burn‐in of 2 × 10^4^ and a thinning interval of 50 to determine a start point for the R‐ and G‐structures. We then ran three Markov chain Monte Carlo (MCMC) chains on each phylogenetic tree for 2400 iterations, discarded the first 400 iterations as burn‐in and sampled every 1000 iterations, for a total posterior sample of 2000 solutions (2 per tree). The effective sample sizes exceeded 1400 for all parameters tested. Chain convergence was assessed using Gelman–Rubin statistic, with potential‐scale reduction values less than 1.1 for all model outputs. The autocorrelation was determined using function ‘autocorr’, with 0.1 used as a target threshold. For each model, we also estimated and reported the ‘conditional’ *R* ^2^ values, that is, the proportion of total variance explained by both the fixed and random effects (Nakagawa & Schielzeth, 2013).

## 3 RESULTS

The final dataset comprised 1451 nest measurement records from 1117 species with complete information on all predictors. In total, 156 species had measurements from both museum specimens and the literature, 256 species had measurements only from museum specimens, and 705 species had measurements only from the literature.

### 3.1 Phylogenetic and geographical distribution of outer nest volume

**FIGURE 2:**
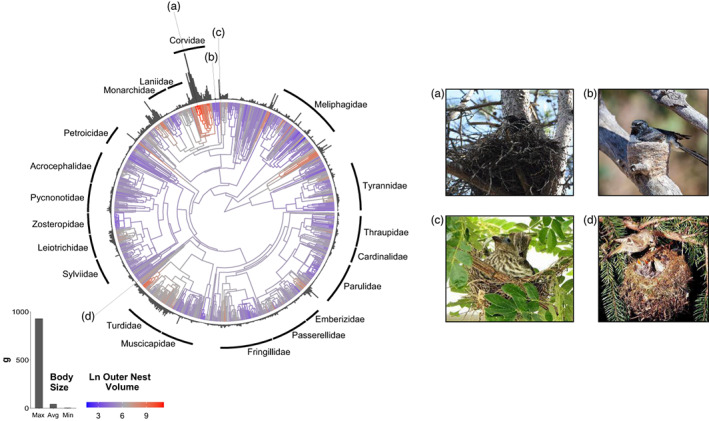
Distribution of outer nest volume (cm^3^, log‐transformed) and body mass (g) in passerines across a single topology from birdtree.org, using the Hackett backbone (Jetz et al., 2012), *n* = 827 species, left panel. The ancestral state reconstructions of nest volume are visualised on the tree structure while the grey bars on the outside represent the corresponding body mass per species. For ease of interpretation, only the names of families with records for 15 species or more have been displayed. Four species are further highlighted in the right panel; all images sourced from the Macaulay Library at the Cornell Lab of Ornithology: (a) *Corvus corax* or common raven (asset ML343861691) builds the largest nest in absolute terms while (b) *Rhipidura leucophrys* or willie wagtail (ML189527291), along with other fantails, has one of the smallest nest volumes; (c) *Sphecotheres vieilloti* or Australasian figbird (ML292333431) builds a relatively small nest for its body mass while (d) *Regulus regulus* or goldcrest (ML712648) has the largest nest with respect to its weight (<6 g).

**FIGURE 3:**
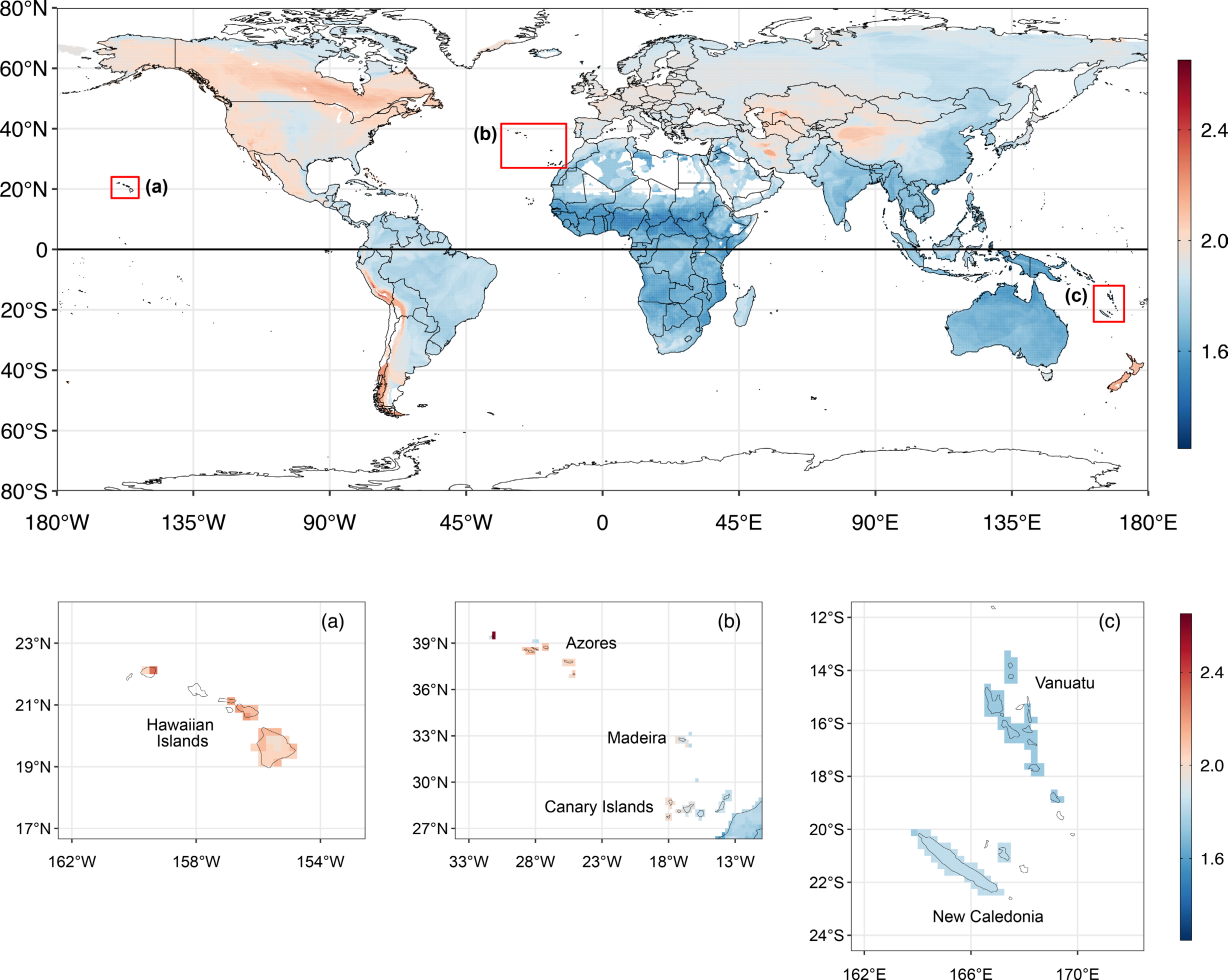
Geographical distribution of the mean outer nest volume divided by body mass per 0.25° grid cell (*n* = 827 species). Grid cells with fewer than four species have been removed from the visualisation and therefore appear blank. The subpanels (a–c) illustrate variation in nest size in different island groups, with all grid cells containing at least one species displayed. The same colour scale has been retained for all panels to ensure comparability; values along the *y*‐ and *x*‐axes correspond to the latitude and longitude, respectively.

The mean reconstructed ancestral nest volume of 353 cm^3^ matches that of the spotted flycatcher *Muscicapa striata*, but this estimate has a large confidence interval [64–2123 cm^3^] that encompasses the majority of species in the dataset. The phylogenetic distribution of outer nest volume and body mass indicates that there is a strong relationship between these two variables, with large‐bodied passerines generally constructing the largest nests (*r* = 0.69, *p* = 0.002, *n* = 827 species; Figure 2). Both variables are also phylogenetically conserved, with Pagel's *λ* values ranging from 0.90 to 0.96 and from 0.95 to 0.99 for log‐transformed values of nest volume and body mass, respectively. In absolute terms, the largest nest in our sample is built by the common raven at 151,586 cm^3^ (*Corvus corax*), but the goldcrest (*Regulus regulus*) constructs the bulkiest nest for its body size (<6 g) at 297 cm^3^. The map of variation in the mean outer nest volume, corrected for body mass, suggests that climate and biogeographical factors may also shape the distribution of nest size on a global scale (Figure 3). We observe a concentration of larger nests in higher latitudes and altitudes, for example, in the area surrounding the Hudson Bay (latitude ~55°N–65°N), and along the Andes and the Himalayas. The difference in nest size between the mainland and some islands provides another contrast, for example, nests are smaller in Australia (here defined as a continent) compared to New Zealand, in southern and eastern Africa compared to Madagascar, or in north‐western Africa compared to the Canary Islands (see Figure 3b).

### 3.2 Drivers of variation in nest volume

**FIGURE 4:**
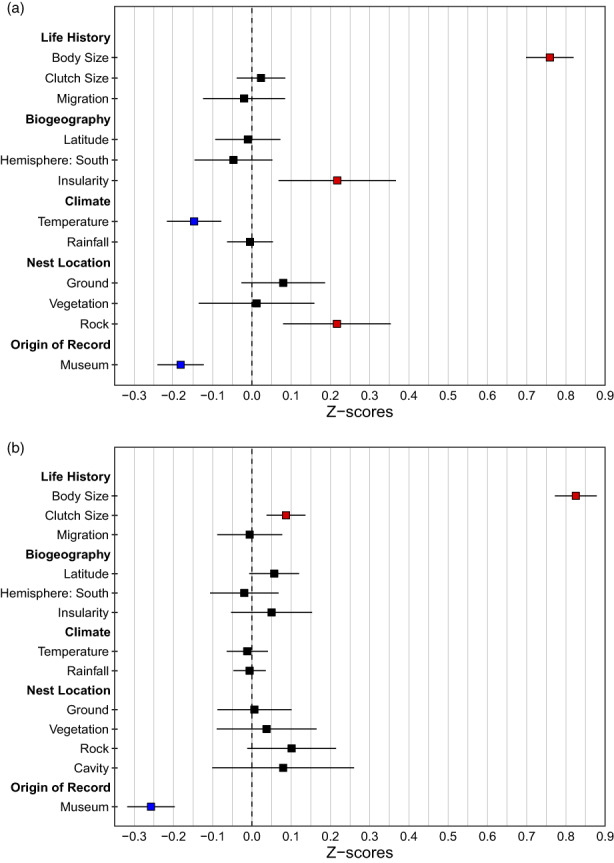
Predictors of (a) outer nest volume (*n* = 1002 records, *n* = 827 species) and (b) inner nest volume (*n* = 1218 records, *n* = 965 species) calculated with a Bayesian phylogenetic mixed model. Significant predictors can be identified by a substantial shift from 0; significant positive and negative associations highlighted in red and blue, respectively. See Tables S3 and S4 for further information.

While all eight types of nest size measurements included in the study are moderately to highly correlated with each other (*r* > 0.4, *p* < 0.001, *n* = 1002 records; Table S2), the output from BPMMs indicates that variation in the outer and inner dimensions of the nest is driven by two different sets of factors. After taking into account variation due to the origin of record, with museum specimens being consistently smaller compared to measurements from the literature, we find that parental body size is the strongest predictor of both outer and inner nest volume: larger birds generally build bulkier nests (*z* = 0.750 and *z* = 0.825, respectively, *p* < 0.001, Figure 4, Tables S3 and S4). In line with evidence from intraspecific studies that nest size increases with lower temperatures, and after controlling for body size, we find across our global sample that nests in colder climates have a greater outer nest volume than nests in warmer climates (*z* = −0.145, *p* < 0.001). We also observe larger nests in island‐dwellers (*z* = 0.209, *p* = 0.001) irrespective of the insularity threshold (*z* = 0.236, *p* = 0.002, Table S5) and in birds that nest on rocks or cliffs (*z* = 0.218, *p* = 0.003), consistent with the expectation that nest size will increase with reduced predation risk. Although inner nest cup volume correlates positively with larger clutch sizes (*z* = 0.087, *p* < 0.001), it is not related to any extrinsic factors (Tables S4 and S6). Additional analyses considering nest height rather than location, however, show that nest placement higher above the ground is associated with smaller volumes of both outer and inner nest cup (*z* = −0.084, *p* = 0.001 and *z* = −0.056, *p* = 0.003, respectively, Tables S7 and S8). The output from models with the other nest dimensions as response variables broadly corroborates these results (Tables S9–S14). In addition to the trends identified above, the external nest diameter is smaller in the southern compared to northern hemisphere (*z* = −0.093, *p* = 0.032, Table S10) while nests built in vegetation have taller outer cups compared to nests placed in other locations (*z* = 0.237, *p* = 0.014, Table S12). Furthermore, internal nest diameter is larger in species that nest on rocks (*z* = 0.141, *p* = 0.010, Table S13) and the body mass of the species is the only significant predictor of the internal nest cup height, with heavier birds constructing taller inner cups (*z* = 0.503, *p* < 0.001, Table S14). The conditional *R* ^2^ values (i.e. the proportion of variance explained by both random and fixed effects) exceed 0.8 for all types of nest dimensions except for internal nest height (0.746, see Tables S3–S14), which suggests low residual or intraspecific variance relative to the differences observed among species. Further analysis using a subset of measurements from museum specimens support this trend, as the estimated repeatability coefficients exceed 0.8 for all types of nest dimensions except for external and internal nest height (see Table S15).

### 3.3 Nest volume as a sexual signal

**FIGURE 5:**
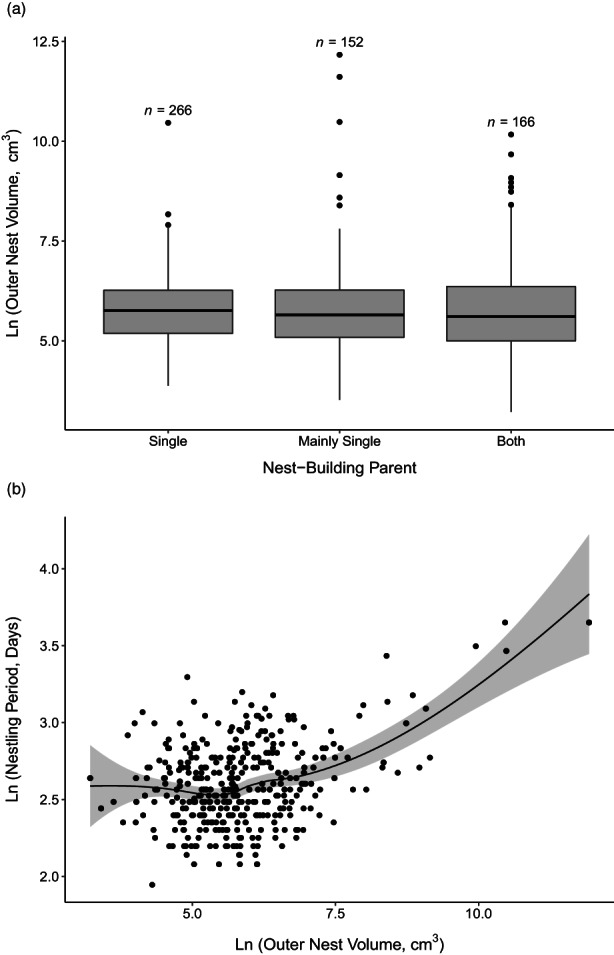
(a) Tukey box and whisker plot of variation in outer nest volume by the number of nest‐building parents (*n* = 584 records, *n* = 434 species). The ends of the grey boxes correspond to the first and third quartiles of data distribution; the line in the middle represents the median value. Whiskers indicate the minimum and maximum values excluding outliers, which are calculated as first and third quartiles ±1.5 times the interquartile range. (b) Relationship between nestling period and outer nest volume, with LOWESS regression line displayed (*n* = 374 species).

We do not find evidence that nest size reflects the number of parents involved in nest‐building or signals differential investment in care for nestlings. Using a smaller dataset of species where the number of parents building the nest is known (*n* = 434) and controlling for phylogeny and other predictors of outer nest size, we show that biparental builders do not build larger nests than female builders (*z* = −0.049, *p* = 0.537, Figure 5a, Table S16). While the scatterplot of raw data implies that nestlings stay longer in nests that have larger outer nest volumes (Figure 5b), the output from the BPMM indicates that larger birds build larger nests and also exhibit longer nestling periods (*z* = 0.411, *p* < 0.001, Table S17). Nest volume is no longer a significant predictor of nestling period after we account for body size and phylogenetic signal (*z* = −0.050, *p* = 0.414).

## 4 DISCUSSION

Our analysis of the global variation in nest size shows that, after accounting for phylogeny, both inner and outer components of the nest structure are strongly correlated with the body size of the species, but the remaining variation is explained by two different sets of factors. While extrinsic drivers, such as temperature and predation threat, appear to be more important in shaping the outer volume of the nest, the inner volume primarily reflects variation in clutch size. We observe clear latitudinal and altitudinal gradients in the outer nest volume, meaning that the pattern of larger or thicker nests in the north of the breeding range or at high elevations—first detected at a population level (e.g. Crossman et al., 2011; Kern & Van Riper, 1984; Mainwaring et al., 2014)—does scale globally and across a multitude of species. Our finding of larger nests in insular species irrespective of island size, or on cliffs or rocks, supports the prediction that reduced levels of predation can lead to increases in nest size. The additional analysis showing that nests get smaller the farther off the ground they are built, however, contradicts this link, perhaps highlighting the role of physical constraints on the size of off‐ground nests or the complex relationship between nest location and predation threat. We also find across our sample that the nest volumes of museum specimens are consistently smaller compared to those sourced from the literature, underscoring the need to consider the origin of data in studies such as these. Finally, while a number of intraspecific studies have documented the use of nest dimensions as a signal of mate quality (e.g. Soler et al., 1995; Tomás et al., 2013), we find no evidence that post‐mating sexual selection is an important driver of nest size investment on a macroevolutionary scale.

Using an extensive dataset of measurements from handbooks and museum specimens, we demonstrate that interspecific variation in passerine nest size is correlated with variation in temperature on a global scale. One potential consequence of this result is that rapid environmental change resulting from global warming might decrease the reproductive output of a species, if individuals construct nests that are suboptimal for altered breeding conditions and thus yield fewer surviving young. The success of the species might therefore hinge on the level of plasticity in its breeding behaviour, that is, the ability of individuals to adjust to changing climate (Mainwaring et al., 2017). Field observations in temperate species suggest that some populations can adapt to different environments by varying their nest structure or materials (e.g. Crossman et al., 2011) or by shifting the start of migration or egg‐laying behaviours (e.g. Charmantier et al., 2008; Smallegange et al., 2010). Experimental studies in zebra finches *Taeniopygia guttata* have shown that reproductive pairs actively alter the composition of their nests in response to local temperature (Campbell et al., 2018; Edwards et al., 2020), thus providing a direct test of plasticity in nest‐building behaviour. Further work is needed to establish the extent to which such plasticity can protect against poor reproductive outcomes across passerine species. Our analysis also demonstrates the value of nest collections—currently an underused resource (Russell et al., 2013)—for studies of large‐scale patterns of variation in avian reproductive traits. In particular, we note that the collection dates of the nests presented here span a century and a half. Tracking change in nest morphology at different time points highlights another potential use of museum collections for researchers interested in the impacts of global warming. Moreover, although we strived to incorporate some degree of intraspecific variation in our analyses, we were unable to obtain a consistently high number of samples per species or to ensure even sampling throughout each species breeding range. We would welcome future studies that explore the impact of within‐species geographical variation on the macroevolutionary relationships studied here.

Our analysis indicates that colder climates are associated with larger volumes of nest material than warmer climates irrespective of the type of material used. Nest dimensions do not vary with the level of rainfall in the breeding environment, however, in contrast to temperature. This could be interpreted as evidence that incubating parents play a more important role in protecting the offspring from excess humidity than does the structure of the nest (Mainwaring et al., 2014). Alternatively, species breeding in areas with high rainfall might preferentially use nest materials with fast drying times such as roots and woody stems (Biddle et al., 2019), which is an aspect of nest morphology not explored in our analysis. A study in 36 Australian cup‐nesting species breeding in different environments showed that temperature and rainfall correlate with the type of material incorporated in the nest more than the nest size because different materials vary in their thermal conductance, that is, the rate heat travels across the nest structure (Heenan et al., 2015). Of commonly used nest materials, for example, down feathers exhibit the best insulating properties while grass performs the worst (Hilton et al., 2004). An assessment of materials incorporated in the museum specimens, for example, by employing non‐invasive techniques where the nest composition is estimated from images of specimens (Sugasawa et al., 2021), in combination with direct estimates of thermal conductance of each nest following Heenan et al. (2015), could provide further insight into the variation in nest properties across different climates.

We here demonstrate that the reduction in predation threat associated with certain nest locations and insularity could be an important macroevolutionary driver of nest size on a global scale. We observe larger nests in species that breed in cliffs or on rocks, which might be a direct consequence of their relative inaccessibility to predators and hence the relaxation of selection for smaller, less visible nests. We also find support for larger nests on islands compared to the mainland which, combined with an observation of longer nestling periods in island‐dwelling species (Cooney et al., 2020), suggests that reproductive traits in birds evolve in a predictable manner in response to the reduced predation threat on islands. Not all of our predator‐related predictions were supported, however. In particular, we find no relationship between nest size and ground‐nesting and show that placing nest higher above ground is associated with a decrease, rather than an increase, in outer nest volume, even though if increased predation leads to reduction in nest size we might expect to find smaller nests on the ground. One potential explanation for this result could be that the level of predation threat on the ground is not uniform and indeed can vary depending on the surrounding vegetation, with on‐ground nesters suffering greater predation rates compared to off‐ground nesters in shrub and grassland habitats and the opposite pattern in forest habitats (Martin, 1993). The decrease in the size of nests farther from the ground could also reflect physical constraints on building large structures in these locations, or increased risk from visually oriented avian predators while ground nests are more frequently preyed on by mammals that use olfactory stimuli (Santisteban et al., 2002; Söderström et al., 1998). In addition, our approach does not allow us to fully separate between the predation risk associated with the body size of the parent—shown to be higher for larger than smaller birds (Biancucci & Martin, 2010)—and the nest size itself. Further studies using direct estimates of nest predation, rather than proxies such as location and insularity, would help elucidate the relationship between nest size and predation.

In contrast to the comparative study in European passerines (Soler et al., 1998), we do not find that species where both parents build produce larger nests compared to female builders, or that nest size is linked to the parental effort in later stages of offspring development. While biparental builders may have a better opportunity to assess the quality of their mate during nest building and also benefit from the additional energetic contribution by the male, this phenomenon does not appear to increase nest volumes across species at the global scale. Furthermore, there is some evidence of differential investment in species where only one parent builds, suggesting that nest volume may provide a reliable estimate of parental effort even if information on nest‐building activity itself is scarce or absent. For example, in blue tits *Cyanistes caeruleus*, where the nest is built exclusively by females, males are more risk‐averse when they provision young in nests that have been experimentally reduced compared to enlarged or control nests—which then negatively affects the reproductive output of the pair (Tomás et al., 2013). As Soler et al. (1998) did not account for environmental conditions in their analysis, it is possible that within their sample the distribution of biparental and female builders was correlated with an underlying factor that also affects nest size, such as lower temperatures.

## 5 CONCLUSIONS

In this study, we provide a broad‐scale assessment of how climate, predation and life‐history traits together shape interspecific variation in nest size, a key reproductive trait of birds. Our work also highlights the value of museum nest collections for macroecological studies and conservation research. Using museum specimens and written records, we demonstrate that macroevolutionary trends in passerine cup nest size are closely tied to parental body size, temperature and the level of predation threat in the breeding range, but not to precipitation or parental investment. This relationship with temperature in particular suggests that nest size and its match or mismatch to the local environment can have a direct impact on the reproductive output of a species. Therefore, the ability to alter nest structure in response to climate conditions could potentially be crucial to species' survival in a rapidly changing world.

## AUTHOR CONTRIBUTIONS

Karina Vanadzina and Catherine Sheard designed the study. Karina Vanadzina, Catherine Sheard and Sally E. Street collected the data, with guidance from Susan D. Healy and Kevin N. Laland. Karina Vanadzina performed the analyses with feedback from Catherine Sheard. Karina Vanadzina wrote the manuscript and all authors contributed to the revisions and gave their final approval for publication.

## CONFLICT OF INTEREST

The authors declare that they have no conflict of interest.

## Supporting information


Appendix S1

## Data Availability

The dataset collated for the purposes of this study is available from Zenodo repository at https://doi.org/10.5281/zenodo.7037356 (Vanadzina et al., 2022).
