# Supplementary material for: Global drivers of variation in cup nest size in passerine birds
Source: J Anim Ecol. 2022 Oct 2;92(2):338–51. doi: 10.1111/1365-2656.13815 (PMC10092846; doi:10.1111/1365-2656.13815)
Supplement: Supplementary file 1 — Appendix S1 [file JANE-92-338-s001.docx]

**Supporting Tables S1–S17**

**Table S1.** Structure of Bayesian phylogenetic mixed models (BPMMs) used for assessing variation I) in the outer and inner nest volume, II) in additional types of nest dimensions, III) in outer nest volume with respect to nest builder identity and in nestling period (i.e., test of differential allocation hypothesis, DAH); sample sizes and references to output Tables in this supplementary document also included. 0/1 signifies a discrete variable with two – absent or present – categories; ‘0’ indicates the reference category for all discrete predictors. Latitude was obtained from coordinates of species’ breeding range midpoint located in either northern or southern (N/S) hemisphere. Insularity score of ‘1’ signifies that more than 90% of species’ range overlaps with landmass shapefiles that are 1) smaller than 2,000,000 km^2^ corresponding to the area of Greenland (Insularity), 2) smaller than 2,000 km^2^ (Insularity_2). For tropical species (species’ range midpoint between 23.5° and -23.5° in latitude), temperature and rainfall signify annual mean temperature and precipitation. For temperate species, these variables signify mean temperature and precipitation from March to June or from September to December (inclusive) for northern (above 23.5° in latitude) and southern (below -23.5° in latitude) hemisphere species, respectively. ‘Outer Volume Full’ corresponds to the outer volume of the nest before the inner cup volume has been subtracted. NA, not applicable.

| **Model** | **Response Variable** | **Predictors** | | | | | | **Records**  ***n*** | **Species**  ***n*** | **Output**  **Table** |
| --- | --- | --- | --- | --- | --- | --- | --- | --- | --- | --- |
|  |  | **Life-History** | **Biogeography** | **Climate** | **Nest Location** | **DAH** | **Origin of Record** |  |  |  |
| **I** | | | | | | | | | | |
| 1 | Outer Volume | Body Size  Clutch Size  Migration: 0/1 | Latitude  Hemisphere:  N (0)/S (1)  Insularity: 0/1 | Temperature  Rainfall | Ground: 0/1  Vegetation: 0/1  Rock: 0/1 | NA | Literature (0)/Museum (1) | 1,002 | 827 | S3 |
| 2 | Inner Volume |  |  |  | Ground: 0/1  Vegetation: 0/1  Rock: 0/1  Cavity: 0/1 |  |  | 1,218 | 965 | S4 |
| 3 | Outer Volume |  | Latitude  Hemisphere:  N (0)/S (1)  Insularity_2: 0/1 |  | Ground: 0/1  Vegetation: 0/1  Rock: 0/1 |  |  | 1,002 | 827 | S5 |
| 4 | Inner Volume |  |  |  | Ground: 0/1  Vegetation: 0/1  Rock: 0/1  Cavity: 0/1 |  |  | 1,218 | 965 | S6 |
| 5 | Outer Volume |  | Latitude  Hemisphere:  N (0)/S (1)  Insularity: 0/1 |  | Nest Height |  |  | 943 | 773 | S7 |
| 6 | Inner Volume |  |  |  | Nest Height |  |  | 1,122 | 883 | S8 |
| **II** | | | | | | | | | | |
| 7 | Outer Volume Full | Body Size  Clutch Size  Migration: 0/1 | Latitude  Hemisphere: N (0)/S (1)  Insularity: 0/1 | Temperature  Rainfall | Ground: 0/1  Vegetation: 0/1  Rock: 0/1 | NA | Literature (0)/Museum (1) | 1,131 | 918 | S9 |
| 8 | External Diameter |  |  |  |  |  |  | 1,279 | 1,014 | S10 |
| 9 | Thickness |  |  |  |  |  |  | 1,129 | 912 | S11 |
| 10 | External Height |  |  |  |  |  |  | 1,157 | 933 | S12 |
| 11 | Internal Diameter |  |  |  | Ground: 0/1  Vegetation: 0/1  Rock: 0/1  Cavity 0/1 |  |  | 1,267 | 1,001 | S13 |
| 12 | Internal Height |  |  |  |  |  |  | 1,274 | 999 | S14 |
| **III** | | | | | | | | | | |
| 13 | Outer Volume | Body Size  Clutch Size  Migration: 0/1 | Latitude  Hemisphere: N (0)/S (1)  Insularity: 0/1 | Temperature  Rainfall | Ground: 0/1  Vegetation: 0/1  Rock: 0/1 | Nest Builder:  Female (0)  Mainly female (1)  Both (2) | Literature (0)/Museum (1) | 584 | 434 | S16 |
| 14 | Nestling Period |  |  |  |  | Outer Volume |  | 374 | 374 | S17 |

**Table S2.** Pearson correlation values (*r*) among nest dimensions in a dataset with all values available (*n* = 1,002 records), with no phylogenetic correction. A value of *r* greater than |0.7| is considered a strong correlation; all correlations are significant (p-value < 0.001). ‘Outer Volume Full’ corresponds to the outer volume of the nest before the inner cup volume has been subtracted.

|  | Outer Volume | Inner Volume | Outer Volume Full | External Diameter | Thickness | External Height | Internal Diameter |
| --- | --- | --- | --- | --- | --- | --- | --- |
| Outer Volume |  |  |  |  |  |  |  |
| Inner Volume | **0.83** |  |  |  |  |  |  |
| Outer Volume Full | **1.00** | **0.85** |  |  |  |  |  |
| External Diameter | **0.73** | **0.83** | **0.74** |  |  |  |  |
| Thickness | **0.74** | **0.75** | **0.74** | **0.97** |  |  |  |
| External Height | 0.60 | **0.77** | 0.61 | **0.75** | 0.69 |  |  |
| Internal Diameter | 0.57 | **0.82** | 0.59 | **0.88** | **0.73** | **0.73** |  |
| Internal Height | 0.44 | **0.71** | 0.46 | 0.67 | 0.58 | **0.79** | **0.70** |

**Table S3.** Predictors of outer nest volume calculated with a BPMM, n = 1,002 records, n = 827 species. Insularity threshold set to islands smaller than 2,000,000 km^2^ corresponding to the area of Greenland. Variables with p-values less than 0.05 are highlighted in bold. CI, credible interval; Var., variance. See Figure 4a (main text) for visualisation.

|  | **Z-score** | **Lower 95% CI** | **Upper 95% CI** | **p-value** |
| --- | --- | --- | --- | --- |
| Intercept | 0.011 | -0.661 | 0.568 | 0.951 |
| **Life History** | | | | |
| Body Size | **0.750** | **0.685** | **0.811** | **<0.001** |
| Clutch Size | 0.019 | -0.045 | 0.077 | 0.572 |
| Migration | -0.027 | -0.136 | 0.076 | 0.629 |
| **Biogeography** | | | | |
| Latitude | -0.003 | -0.091 | 0.073 | 0.935 |
| Hemisphere: South | -0.039 | -0.139 | 0.054 | 0.449 |
| Insularity | **0.209** | **0.088** | **0.348** | **0.001** |
| **Climate** | | | | |
| Temperature | **-0.145** | **-0.213** | **-0.078** | **<0.001** |
| Rainfall | -0.011 | -0.062 | 0.048 | 0.673 |
| **Nest Location** | | | | |
| Ground | 0.076 | -0.026 | 0.190 | 0.173 |
| Vegetation | 0.032 | -0.120 | 0.201 | 0.707 |
| Rock | **0.218** | **0.076** | **0.366** | **0.003** |
| **Origin of Record** | | | | |
| Museum | **-0.178** | **-0.247** | **-0.109** | **<0.001** |
|  |  |  |  |  |
| Phylogeny | 0.531 | 0.363 | 0.705 |  |
| Between-species Var. | 0.037 | 0.005 | 0.071 |  |
| Within-species or Residual Var. | 0.111 | 0.086 | 0.133 |  |
|  |  |  |  |  |
| Conditional R^2^ | 0.913 |  |  |  |

**Table S4.** Predictors of inner nest volume calculated with a BPMM, n = 1,218 records, n = 965 species. Insularity threshold set to islands smaller than 2,000,000 km^2^ corresponding to the area of Greenland. Variables with p-values less than 0.05 are highlighted in bold. CI, credible interval; Var., variance. See Figure 4b (main text) for visualisation.

|  | **Z-score** | **Lower 95% CI** | **Upper 95% CI** | **p-value** |
| --- | --- | --- | --- | --- |
| Intercept | 0.038 | -0.377 | 0.430 | 0.852 |
| **Life History** | | | | |
| Body Size | **0.825** | **0.778** | **0.879** | **<0.001** |
| Clutch Size | **0.087** | **0.036** | **0.136** | **<0.001** |
| Migration | -0.006 | -0.096 | 0.077 | 0.868 |
| **Biogeography** | | | | |
| Latitude | 0.057 | -0.011 | 0.120 | 0.085 |
| Hemisphere: South | -0.020 | -0.102 | 0.068 | 0.640 |
| Insularity | 0.050 | -0.062 | 0.153 | 0.362 |
| **Climate** | | | | |
| Temperature | -0.012 | -0.069 | 0.040 | 0.684 |
| Rainfall | -0.006 | -0.050 | 0.035 | 0.780 |
| **Nest Location** | | | | |
| Ground | 0.006 | -0.077 | 0.101 | 0.902 |
| Vegetation | 0.037 | -0.069 | 0.164 | 0.563 |
| Rock | 0.101 | -0.004 | 0.214 | 0.067 |
| Cavity | 0.079 | -0.107 | 0.260 | 0.387 |
| **Origin of Record** | | | | |
| Museum | **-0.257** | **-0.315** | **-0.197** | **<0.001** |
|  |  |  |  |  |
| Phylogeny | 0.249 | 0.171 | 0.331 |  |
| Between-species Var. | 0.011 | 0.002 | 0.023 |  |
| Within-species or Residual Var. | 0.144 | 0.126 | 0.162 |  |
|  |  |  |  |  |
| Conditional R^2^ | 0.869 |  |  |  |

**Table S5.** Predictors of outer nest volume calculated with a BPMM, n = 1,002 records, n = 827 species. Insularity threshold set to islands smaller than 2,000 km^2^. Variables with p-values less than 0.05 are highlighted in bold. CI, credible interval; Var., variance.

|  | **Z-score** | **Lower 95% CI** | **Upper 95% CI** | **p-value** |
| --- | --- | --- | --- | --- |
| Intercept | 0.015 | -0.659 | 0.564 | 0.942 |
| **Life History** | | | | |
| Body Size | **0.751** | **0.686** | **0.812** | **<0.001** |
| Clutch Size | 0.019 | -0.045 | 0.077 | 0.549 |
| Migration | -0.031 | -0.131 | 0.082 | 0.579 |
| **Biogeography** | | | | |
| Latitude | -0.005 | -0.087 | 0.075 | 0.914 |
| Hemisphere: South | -0.023 | -0.121 | 0.072 | 0.651 |
| Insularity_2 | **0.236** | **0.076** | **0.381** | **0.002** |
| **Climate** | | | | |
| Temperature | **-0.159** | **-0.230** | **-0.094** | **<0.001** |
| Rainfall | 0.013 | -0.034 | 0.068 | 0.657 |
| **Nest Location** | | | | |
| Ground | 0.083 | -0.020 | 0.195 | 0.132 |
| Vegetation | 0.030 | -0.131 | 0.188 | 0.731 |
| Rock | **0.219** | **0.080** | **0.370** | **0.002** |
| **Origin of Record** | | | | |
| Museum | **-0.177** | **-0.244** | **-0.107** | **<0.001** |
|  |  |  |  |  |
| Phylogeny | 0.529 | 0.359 | 0.703 |  |
| Between-species Var. | 0.038 | 0.005 | 0.072 |  |
| Within-species or Residual Var. | 0.111 | 0.087 | 0.134 |  |
|  |  |  |  |  |
| Conditional R^2^ | 0.913 |  |  |  |

**Table S6.** Predictors of inner nest volume calculated with a BPMM, n = 1,218 records, n = 965 species. Insularity threshold set to islands smaller than 2,000 km^2^.Variables with p-values less than 0.05 are highlighted in bold. CI, credible interval; Var., variance.

|  | **Z-score** | **Lower 95% CI** | **Upper 95% CI** | **p-value** |
| --- | --- | --- | --- | --- |
| Intercept | 0.030 | -0.364 | 0.452 | 0.892 |
| **Life History** | | | | |
| Body Size | **0.826** | **0.778** | **0.873** | **<0.001** |
| Clutch Size | **0.090** | **0.034** | **0.138** | **<0.001** |
| Migration | 0.001 | -0.078 | 0.086 | 0.955 |
| **Biogeography** | | | | |
| Latitude | 0.052 | -0.010 | 0.116 | 0.106 |
| Hemisphere: South | -0.015 | -0.098 | 0.069 | 0.724 |
| Insularity_2 | 0.119 | -0.023 | 0.240 | 0.073 |
| **Climate** | | | | |
| Temperature | -0.018 | -0.075 | 0.035 | 0.504 |
| Rainfall | -0.002 | -0.047 | 0.038 | 0.92 |
| **Nest Location** | | | | |
| Ground | 0.008 | -0.083 | 0.096 | 0.854 |
| Vegetation | 0.042 | -0.066 | 0.160 | 0.468 |
| Rock | 0.105 | -0.0004 | 0.210 | 0.051 |
| Cavity | 0.082 | -0.091 | 0.272 | 0.387 |
| **Origin of Record** | | | | |
| Museum | **-0.256** | **-0.316** | **-0.196** | **<0.001** |
|  |  |  |  |  |
| Phylogeny | 0.245 | 0.175 | 0.331 |  |
| Between-species Var. | 0.011 | 0.002 | 0.023 |  |
| Within-species or Residual Var. | 0.144 | 0.126 | 0.162 |  |
|  |  |  |  |  |
| Conditional R^2^ | 0.868 |  |  |  |

**Table S7.** Predictors of outer nest volume calculated with a BPMM, n = 943 records, n = 773 species; the mean height of nest placement from the ground included as a predictor. Insularity threshold set to islands smaller than 2,000,000 km^2^ corresponding to the area of Greenland. Variables with p-values less than 0.05 are highlighted in bold. CI, credible interval; Var., variance.

|  | **Z-score** | **Lower 95% CI** | **Upper 95% CI** | **p-value** |
| --- | --- | --- | --- | --- |
| Intercept | 0.039 | -0.538 | 0.565 | 0.882 |
| **Life History** | | | | |
| Body Size | **0.784** | **0.722** | **0.851** | **<0.001** |
| Clutch Size | 0.030 | -0.032 | 0.099 | 0.375 |
| Migration | -0.028 | -0.130 | 0.091 | 0.628 |
| **Biogeography** | | | | |
| Latitude | 0.012 | -0.073 | 0.088 | 0.813 |
| Hemisphere: South | -0.039 | -0.146 | 0.066 | 0.448 |
| Insularity | **0.196** | **0.062** | **0.328** | **0.002** |
| **Climate** | | | | |
| Temperature | **-0.143** | **-0.213** | **-0.077** | **<0.001** |
| Rainfall | 0.008 | -0.050 | 0.059 | 0.756 |
| **Nest Location** | | | | |
| Nest Height | **-0.084** | **-0.129** | **-0.038** | **0.001** |
| **Origin of Record** | | | | |
| Museum | **-0.185** | **-0.250** | **-0.117** | **<0.001** |
|  |  |  |  |  |
| Phylogeny | 0.511 | 0.341 | 0.688 |  |
| Between-species Var. | 0.040 | 0.003 | 0.072 |  |
| Within-species or Residual Var. | 0.105 | 0.084 | 0.130 |  |
|  |  |  |  |  |
| Conditional R^2^ | 0.917 |  |  |  |

**Table S8**. Predictors of inner nest volume calculated with a BPMM, n = 1,122 records, n = 883 species; the mean height of nest placement from the ground included as a predictor. Insularity threshold set to smaller than 2,000,000 km^2^ corresponding to the area of Greenland. Variables with p-values less than 0.05 are highlighted in bold. CI, credible interval; Var., variance.

|  | **Z-score** | **Lower 95% CI** | **Upper 95% CI** | **p-value** |
| --- | --- | --- | --- | --- |
| Intercept | 0.084 | -0.274 | 0.463 | 0.657 |
| **Life History** | | | | |
| Body Size | **0.846** | **0.796** | **0.893** | **<0.001** |
| Clutch Size | **0.087** | **0.034** | **0.140** | **<0.001** |
| Migration | -0.002 | -0.093 | 0.086 | 0.980 |
| **Biogeography** | | | | |
| Latitude | **0.076** | **0.007** | **0.144** | **0.031** |
| Hemisphere: South | -0.046 | -0.128 | 0.043 | 0.293 |
| Insularity | 0.080 | -0.034 | 0.193 | 0.169 |
| **Climate** | | | | |
| Temperature | 0.010 | -0.052 | 0.062 | 0.721 |
| Rainfall | -0.006 | -0.049 | 0.038 | 0.797 |
| **Nest Location** | | | | |
| Nest Height | **-0.056** | **-0.090** | **-0.023** | **0.003** |
| **Origin of Record** | | | | |
| Museum | **-0.254** | **-0.314** | **-0.191** | **<0.001** |
|  |  |  |  |  |
| Phylogeny | 0.220 | 0.148 | 0.303 |  |
| Between-species Var. | 0.011 | 0.002 | 0.024 |  |
| Within-species or Residual Var. | 0.137 | 0.119 | 0.157 |  |
|  |  |  |  |  |
| Conditional R^2^ | 0.871 |  |  |  |

**Table S9.** Predictors of outer nest volume (in full, i.e., inner volume not subtracted) calculated with a BPMM, n = 1,131 records, n = 918 species. Insularity threshold set to smaller than 2,000,000 km^2^ corresponding to the area of Greenland. Variables with p-values less than 0.05 are highlighted in bold. CI, credible interval; Var., variance.

|  | **Z-score** | **Lower 95% CI** | **Upper 95% CI** | **p-value** |
| --- | --- | --- | --- | --- |
| Intercept | -0.126 | -0.633 | 0.390 | 0.638 |
| **Life History** | | | | |
| Body Size | **0.759** | **0.700** | **0.817** | **<0.001** |
| Clutch Size | 0.044 | -0.006 | 0.110 | 0.128 |
| Migration | -0.003 | -0.092 | 0.096 | 0.948 |
| **Biogeography** | | | | |
| Latitude | -0.019 | -0.091 | 0.050 | 0.613 |
| Hemisphere: South | -0.050 | -0.126 | 0.046 | 0.259 |
| Insularity | **0.198** | **0.074** | **0.312** | **0.003** |
| **Climate** | | | | |
| Temperature | **-0.138** | **-0.200** | **-0.080** | **<0.001** |
| Rainfall | -0.016 | -0.063 | 0.033 | 0.518 |
| **Nest Location** | | | | |
| Ground | 0.018 | -0.080 | 0.122 | 0.744 |
| Vegetation | 0.096 | -0.050 | 0.246 | 0.222 |
| Rock | **0.216** | **0.091** | **0.354** | **0.005** |
| **Origin of Record** | | | | |
| Museum | **-0.151** | **-0.210** | **-0.089** | **<0.001** |
|  |  |  |  |  |
| Phylogeny | 0.426 | 0.283 | 0.555 |  |
| Between-species Var. | 0.037 | 0.009 | 0.068 |  |
| Within-species or Residual Var. | 0.109 | 0.090 | 0.130 |  |
|  |  |  |  |  |
| Conditional R^2^ | 0.906 |  |  |  |

**Table S10.** Predictors of external nest diameter calculated with a BPMM, n = 1,279 records, n = 1,014 species. Insularity threshold set to smaller than 2,000,000 km^2^ corresponding to the area of Greenland. Variables with p-values less than 0.05 are highlighted in bold. CI, credible interval; Var., variance.

|  | **Z-score** | **Lower 95% CI** | **Upper 95% CI** | **p-value** |
| --- | --- | --- | --- | --- |
| Intercept | -0.109 | -0.531 | 0.352 | 0.625 |
| **Life History** | | | | |
| Body Size | **0.808** | **0.754** | **0.856** | **<0.001** |
| Clutch Size | 0.045 | -0.008 | 0.100 | 0.100 |
| Migration | 0.044 | -0.042 | 0.140 | 0.345 |
| **Biogeography** | | | | |
| Latitude | -0.037 | -0.113 | 0.031 | 0.282 |
| Hemisphere: South | **-0.093** | **-0.179** | **-0.010** | **0.032** |
| Insularity | **0.164** | **0.054** | **0.282** | **0.005** |
| **Climate** | | | | |
| Temperature | **-0.114** | **-0.168** | **-0.053** | **<0.001** |
| Rainfall | -0.047 | -0.095 | 0.003 | 0.062 |
| **Nest Location** | | | | |
| Ground | 0.063 | -0.029 | 0.158 | 0.185 |
| Vegetation | 0.033 | -0.100 | 0.172 | 0.651 |
| Rock | **0.168** | **0.055** | **0.301** | **0.005** |
| **Origin of Record** | | | | |
| Museum | 0.026 | -0.031 | 0.084 | 0.393 |
|  |  |  |  |  |
| Phylogeny | 0.308 | 0.208 | 0.418 |  |
| Between-species Var. | 0.037 | 0.008 | 0.066 |  |
| Within-species or Residual Var. | 0.135 | 0.114 | 0.159 |  |
|  |  |  |  |  |
| Conditional R^2^ | 0.883 |  |  |  |

**Table S11.** Predictors of nest thickness calculated with a BPMM, n = 1,129 records, n = 912 species. Insularity threshold set to smaller than 2,000,000 km^2^ corresponding to the area of Greenland. Variables with p-values less than 0.05 are highlighted in bold. CI, credible interval; Var., variance.

|  | **Z-score** | **Lower 95% CI** | **Upper 95% CI** | **p-value** |
| --- | --- | --- | --- | --- |
| Intercept | -0.303 | -0.941 | 0.412 | 0.370 |
| Life History | | | | |
| Body Size | **0.617** | **0.546** | **0.695** | **<0.001** |
| Clutch Size | -0.023 | -0.094 | 0.059 | 0.562 |
| Migration | 0.052 | -0.074 | 0.204 | 0.442 |
| **Biogeography** | | | | |
| Latitude | -0.029 | -0.123 | 0.078 | 0.561 |
| Hemisphere: South | -0.040 | -0.163 | 0.093 | 0.550 |
| Insularity | **0.183** | **0.029** | **0.362** | **0.026** |
| **Climate** | | | | |
| Temperature | **-0.159** | **-0.240** | **-0.072** | **<0.001** |
| Rainfall | -0.023 | -0.093 | 0.041 | 0.519 |
| **Nest Location** | | | | |
| Ground | 0.127 | -0.022 | 0.261 | 0.085 |
| Vegetation | 0.077 | -0.131 | 0.267 | 0.443 |
| Rock | **0.193** | **0.010** | **0.385** | **0.049** |
| **Origin of Record** | | | | |
| Museum | **0.124** | **0.037** | **0.215** | **0.011** |
|  |  |  |  |  |
| Phylogeny | 0.669 | 0.429 | 0.924 |  |
| Between-species Var. | 0.056 | 0.004 | 0.113 |  |
| Within-species or Residual Var. | 0.259 | 0.211 | 0.306 |  |
|  |  |  |  |  |
| Conditional R^2^ | 0.814 |  |  |  |

**Table S12.** Predictors of external nest height calculated with a BPMM, n = 1,157 records, n = 933 species. Insularity threshold set to smaller than 2,000,000 km^2^ corresponding to the area of Greenland. Variables with p-values less than 0.05 are highlighted in bold. CI, credible interval; Var., variance.

|  | **Z-score** | **Lower 95% CI** | **Upper 95% CI** | **p-value** |
| --- | --- | --- | --- | --- |
| Intercept | -0.117 | -0.885 | 0.560 | 0.750 |
| **Life History** | | | | |
| Body Size | **0.506** | **0.431** | **0.583** | **<0.001** |
| Clutch Size | 0.059 | -0.018 | 0.136 | 0.142 |
| Migration | -0.073 | -0.203 | 0.071 | 0.287 |
| **Biogeography** | | | | |
| Latitude | 0.001 | -0.098 | 0.095 | 0.984 |
| Hemisphere: South | 0.007 | -0.120 | 0.135 | 0.918 |
| Insularity | **0.255** | **0.085** | **0.424** | **0.001** |
| **Climate** | | | | |
| Temperature | **-0.144** | **-0.232** | **-0.065** | **<0.001** |
| Rainfall | 0.037 | -0.026 | 0.110 | 0.296 |
| **Nest Location** | | | | |
| Ground | -0.091 | -0.235 | 0.047 | 0.211 |
| Vegetation | **0.237** | **0.047** | **0.439** | **0.014** |
| Rock | 0.182 | -0.014 | 0.358 | 0.055 |
| **Origin of Record** | | | | |
| Museum | **-0.491** | **-0.577** | **-0.395** | **<0.001** |
|  |  |  |  |  |
| Phylogeny | 0.717 | 0.502 | 0.970 |  |
| Between-species Var. | 0.047 | 0.002 | 0.098 |  |
| Within-species or Residual Var. | 0.272 | 0.224 | 0.319 |  |
|  |  |  |  |  |
| Conditional R^2^ | 0.800 |  |  |  |

**Table S13**. Predictors of inner nest diameter calculated with a BPMM, n = 1,267 records, n = 1,001 species. Insularity threshold set to smaller than 2,000,000 km^2^ corresponding to the area of Greenland. Variables with p-values less than 0.05 are highlighted in bold. CI, credible interval; Var., variance.

|  | **Z-score** | **Lower 95% CI** | **Upper 95% CI** | **p-value** |
| --- | --- | --- | --- | --- |
| Intercept | 0.110 | -0.191 | 0.412 | 0.456 |
| **Life History** | | | | |
| Body Size | **0.863** | **0.825** | **0.903** | **<0.001** |
| Clutch Size | **0.111** | **0.062** | **0.156** | **<0.001** |
| Migration | -0.017 | -0.096 | 0.054 | 0.678 |
| **Biogeography** | | | | |
| Latitude | 0.021 | -0.038 | 0.079 | 0.510 |
| Hemisphere: South | -0.020 | -0.097 | 0.053 | 0.612 |
| Insularity | 0.030 | -0.066 | 0.135 | 0.548 |
| **Climate** | | | | |
| Temperature | -0.018 | -0.067 | 0.033 | 0.459 |
| Rainfall | -0.014 | -0.054 | 0.027 | 0.468 |
| **Nest Location** | | | | |
| Ground | 0.003 | -0.079 | 0.089 | 0.945 |
| Vegetation | -0.036 | -0.143 | 0.080 | 0.522 |
| Rock | **0.141** | **0.048** | **0.245** | **0.010** |
| Cavity | 0.073 | -0.096 | 0.222 | 0.380 |
| **Origin of Record** | | | | |
| Museum | **-0.195** | **-0.254** | **-0.143** | **<0.001** |
|  |  |  |  |  |
| Phylogeny | 0.119 | 0.074 | 0.171 |  |
| Between-species Var. | 0.010 | 0.002 | 0.022 |  |
| Within-species or Residual Var. | 0.151 | 0.136 | 0.169 |  |
|  |  |  |  |  |
| Conditional R^2^ | 0.853 |  |  |  |

**Table S14**. Predictors of internal nest height calculated with a BPMM, n = 1,274 records, n = 999 species. Insularity threshold set to smaller than 2,000,000 km^2^ corresponding to the area of Greenland. Variables with p-values less than 0.05 are highlighted in bold. CI, credible interval; Var., variance.

|  | **Z-score** | **Lower 95% CI** | **Upper 95% CI** | **p-value** |
| --- | --- | --- | --- | --- |
| Intercept | -0.164 | -0.853 | 0.551 | 0.643 |
| **Life History** | | | | |
| Body Size | **0.503** | **0.429** | **0.581** | **<0.001** |
| Clutch Size | 0.035 | -0.051 | 0.119 | 0.428 |
| Migration | 0.031 | -0.104 | 0.167 | 0.676 |
| **Biogeography** | | | | |
| Latitude | 0.066 | -0.035 | 0.169 | 0.207 |
| Hemisphere: South | -0.026 | -0.167 | 0.110 | 0.715 |
| Insularity | 0.079 | -0.083 | 0.266 | 0.361 |
| **Climate** | | | | |
| Temperature | -0.035 | -0.115 | 0.051 | 0.421 |
| Rainfall | -0.003 | -0.068 | 0.072 | 0.928 |
| **Nest Location** | | | | |
| Ground | -0.012 | -0.158 | 0.126 | 0.892 |
| Vegetation | 0.160 | -0.034 | 0.350 | 0.115 |
| Rock | -0.005 | -0.187 | 0.155 | 0.941 |
| Cavity | 0.091 | -0.191 | 0.359 | 0.535 |
| **Origin of Record** | | | | |
| Museum | **-0.265** | **-0.354** | **-0.174** | **<0.001** |
|  |  |  |  |  |
| Phylogeny | 0.768 | 0.532 | 0.996 |  |
| Between-species Var. | 0.025 | 0.003 | 0.062 |  |
| Within-species or Residual Var. | 0.361 | 0.315 | 0.413 |  |
|  |  |  |  |  |
| Conditional R^2^ | 0.746 |  |  |  |

**Table S15.** Repeatability coefficients, i.e., the proportion of the total variance attributable to between-species differences, for all types of nest dimensions in the subset of 1,401 museum specimens from 435 species (Natural History Museum, UK). ‘Outer Volume Full’ corresponds to the outer volume of the nest before the inner cup volume has been subtracted; CI, credible interval.

|  | **Repeatability**  **coefficient** | **Lower 95% CI** | **Upper 95% CI** |
| --- | --- | --- | --- |
| Outer Volume | 0.872 | 0.832 | 0.910 |
| Inner Volume | 0.897 | 0.868 | 0.920 |
| Outer Volume Full | 0.898 | 0.866 | 0.925 |
| External Diameter | 0.910 | 0.886 | 0.931 |
| Thickness | 0.802 | 0.749 | 0.852 |
| External Height | 0.724 | 0.652 | 0.796 |
| Internal Diameter | 0.895 | 0.862 | 0.923 |
| Internal Height | 0.699 | 0.615 | 0.771 |

**Table S16.** Predictors of outer nest volume calculated with a BPMM, with the identify of builder included as a categorical variable; nest building by female is the reference category (n = 584 records, n = 434 species). Insularity threshold set to smaller than 2,000,000 km^2^ corresponding to the area of Greenland. Variables with p-values less than 0.05 are highlighted in bold. CI, credible interval. Cooperative breeders and species where all care is provided by a single female were excluded from the analysis (n = 86); species where nest was built by mainly or exclusively by male were also not included (n = 8). Var., variance.

|  | **Z-score** | **Lower 95% CI** | **Upper 95% CI** | **p-value** |
| --- | --- | --- | --- | --- |
| Intercept | 0.193 | -0.420 | 0.748 | 0.503 |
| **Life History** | | | | |
| Body Size | **0.780** | **0.701** | **0.854** | **<0.001** |
| Clutch Size | 0.058 | -0.024 | 0.136 | 0.165 |
| Migration | -0.115 | -0.263 | 0.020 | 0.110 |
| **Biogeography** | | | | |
| Latitude | -0.003 | -0.108 | 0.102 | 0.940 |
| Hemisphere: South | -0.178 | -0.339 | 0.009 | 0.051 |
| Insularity | 0.125 | -0.072 | 0.326 | 0.222 |
| **Climate** | | | | |
| Temperature | **-0.117** | **-0.209** | **-0.022** | **0.019** |
| Rainfall | -0.012 | -0.073 | 0.053 | 0.709 |
| **Nest Location** | | | | |
| Ground | 0.085 | -0.048 | 0.230 | 0.228 |
| Vegetation | 0.031 | -0.183 | 0.224 | 0.735 |
| Rock | **0.269** | **0.105** | **0.437** | **0.003** |
| **Nest Builder** | | | | |
| Mainly Female | -0.029 | -0.175 | 0.114 | 0.707 |
| Both | -0.049 | -0.200 | 0.101 | 0.537 |
| **Origin of Record** | | | | |
| Museum | **-0.159** | **-0.238** | **-0.075** | **<0.001** |
|  |  |  |  |  |
| Phylogeny | 0.486 | 0.320 | 0.683 |  |
| Between-species Var. | 0.030 | 0.003 | 0.062 |  |
| Within-species or Residual Var. | 0.120 | 0.096 | 0.146 |  |
|  |  |  |  |  |
| Conditional R^2^ | 0.910 |  |  |  |

**Table S17.** Predictors of nestling period calculated with a BPMM, with the average outer nest volume per species included as a predictor in the dataset where the nest-builder identify is known (n = 374 species). Insularity threshold set to smaller than 2,000,000 km^2^ corresponding to the area of Greenland. Variables with p-values less than 0.05 are highlighted in bold. CI, credible interval. Cooperative breeders and species where all care is provided by a single parent were excluded from the analysis (n = 86); species where nest was built by mainly or exclusively by male were also not included (n = 8). Var., variance.

|  | **Z-score** | **Lower 95% CI** | **Upper 95% CI** | **p-value** |
| --- | --- | --- | --- | --- |
| Intercept | 0.420 | -0.639 | 1.347 | 0.407 |
| **Life History** | | | | |
| Body Size | **0.411** | **0.262** | **0.536** | **<0.001** |
| Clutch Size | -0.048 | -0.140 | 0.061 | 0.358 |
| Migration | -0.118 | -0.274 | 0.051 | 0.159 |
| **Biogeography** | | | | |
| Latitude | **-0.138** | **-0.267** | **-0.010** | **0.035** |
| Hemisphere: South | 0.015 | -0.186 | 0.205 | 0.867 |
| Insularity | **0.266** | **0.034** | **0.509** | **0.026** |
| **Climate** | | | | |
| Temperature | -0.094 | -0.211 | 0.018 | 0.114 |
| Rainfall | -0.053 | -0.136 | 0.016 | 0.156 |
| **Nest Location** | | | | |
| Ground | **-0.241** | **-0.394** | **-0.080** | **0.003** |
| Vegetation | -0.215 | -0.420 | 0.003 | 0.055 |
| Rock | 0.075 | -0.098 | 0.252 | 0.388 |
| **Nest Size** | | | | |
| Outer Nest Volume | -0.050 | -0.163 | 0.076 | 0.414 |
|  |  |  |  |  |
| Phylogeny | 1.313 | 0.953 | 1.696 |  |
| Residual Var. | 0.046 | 0.009 | 0.083 |  |
|  |  |  |  |  |
| Conditional R^2^ | 0.971 |  |  |  |
